# Cost-effectiveness of edaravone dexborneol sublingual tablet versus concentrated solution for injection for the treatment of acute ischemic stroke in China

**DOI:** 10.3389/fphar.2025.1661581

**Published:** 2025-11-13

**Authors:** Fan Xie, Yuqiong Lu, Zhuo Yang, Yanlei Zhang, Yujin Bao, Yun Lu

**Affiliations:** 1 School of International Pharmaceutical Business, China Pharmaceutical University, Nanjing, China; 2 State Key Laboratory of Neurology and Oncology Drug Development, Nanjing, China

**Keywords:** cost-effectiveness, edaravone dexborneol, sublingual tablets, acute ischemic stroke, China

## Abstract

**Background:**

Edaravone dexborneol sublingual tablet (EDSL) is a novel therapy for acute ischemic stroke (AIS), proven effective and safe. Its convenient dosage form also provides an alternative administration option, helping to enhance treatment accessibility. This study evaluates the cost-effectiveness of EDSL versus edaravone dexborneol concentrated solution for injection (EDCSI) from the Chinese healthcare system perspective.

**Methods:**

A combination of the short-term decision tree and long-term Markov model was constructed. Clinical data were derived from the TASTE-SL and TASTE trials. To adjust for baseline characteristics between the two treatment groups, stabilized inverse probability of treatment weighting (sIPTW) was applied, and both Average Treatment effect on the Treated (ATT) and Average Treatment Effect (ATE) weighted results were calculated to enhance comparability. Model parameters were extracted from published literature, public databases, and expert interviews. Sensitivity and scenario analyses were conducted to demonstrate the robustness of the base-case results.

**Results:**

There was no statistically significant difference in the proportion of patients achieving mRS score ≤1 between the EDSL and EDCSI groups after sIPTW (ATT: 71.8% vs. 67.6%; ATE: 69.3% vs. 64.6%). Notably, EDSL showed clear cost-effectiveness advantages, with an incremental cost-effectiveness ratio (ICER) of -¥2,089.84 per QALY gained. All sensitivity and scenario analyses confirmed the robustness of the base-case findings.

**Conclusion:**

EDSL represents a more cost-effective therapy for AIS patients compared to EDCSI, offering comparable efficacy at a lower drug cost. A full 14-day treatment course may help maximize patient benefits.

## 1 Introduction

Stroke is an acute neurological disorder caused by cerebrovascular injury and represents a significant health burden domestically and internationally. The 2019 Global Burden of Disease study showed 3.94 million new stroke cases in China that year, out of 12.2 million globally (GBD, 2019 Stroke Collaborators, 2021). Among Chinese patients, the 3-month post-stroke disability rate ranges from 14.6% to 23.1%, the mortality rate from 1.5% to 3.2%, and the recurrence rate is 6.5%. At 1-year follow-up, the disability rate ranges from 13.9% to 14.2%, mortality rate from 3.4% to 6.0% and recurrence rate reaches 10.3% (Chinese Society of Neurology et al., 2024). Acute ischemic stroke (AIS), the most common subtype, comprises roughly 70% of all strokes (Liu et al., 2023a). It can severely impair patients’ daily function and quality of life (Xie et al., 2024), and a study by Peng C (2022) reported that the total medical expenses related to AIS in China reached ¥41.7 billion in 2021 (Peng et al., 2022), imposing a substantial economic burden on patients, families, and society.

Despite continuous improvements in AIS care, significant unmet needs remain in China. First, there is often a considerable delay between AIS onset and hospital admission, especially in remote or rural areas where access to stroke centers is limited. As a result, some patients may miss the critical “golden window” for treatment (Yuan et al., 2023; Yuan et al., 2023) reported that the proportion of patients arriving at the hospital in a timely manner after stroke onset is significantly lower in rural and underserved regions. Second, under payment models such as Diagnosis-Related Groups (DRGs) and Diagnosis-Intervention Packets (DIPs), hospitals often shorten the average length of stay for AIS patients to reduce costs. As a result, patients are frequently discharged before completing the full course of treatment. Wang et al. (2025), Wang Y. et al. (2025) discovered that the mean length of stay diminished from approximately 8 days prior to DRG implementation to approximately 5 days subsequently. Without adequate, timely, and standardized therapy, many experience worse prognoses, higher readmission rates, and prolonged disability, thereby increasing both medical resource consumption and caregiver burden.

In response to these challenges, the edaravone dexborneol sublingual tablet (EDSL) was approved in China in 2024. This formulation offers distinct advantages such as rapid sublingual absorption, avoidance of first-pass hepatic metabolism, and convenient out-of-hospital administration, making it particularly suitable for ensuring treatment continuity after early discharge. The efficacy and safety of EDSL have been further validated in the TASTE-SL trial (NCT04950920) (Fu et al., 2024). Notably, the sublingual tablet formulation of Edaravone Dexborneol (EDSL) is the first innovative stroke therapy worldwide to receive the “Breakthrough Therapy” designation from the U.S. Food and Drug Administration (FDA) and has been granted a Chinese invention patent (Patent No. CN109906077B). This designation is awarded to drugs that demonstrate substantial improvement over available therapies for treating serious or life-threatening conditions. Such recognition validates the scientific innovation and therapeutic potential of this sublingual formulation, highlights the significant clinical value of EDSL, enhances its global relevance, and demonstrates its important role in addressing unmet medical needs in acute stroke treatment.

Given that EDSL has established clinical efficacy and safety, this study aims to further evaluate its cost-effectiveness in Chinese AIS patients. Edaravone dexborneol concentrated solution for injection (EDCSI) was selected as the comparator for several reasons. First, EDCSI and EDSL share the same active ingredients, edaravone and (+)-borneol, which act through dual mechanisms of free radical scavenging and anti-inflammatory effects. Second, EDCSI is the only cytoprotective therapy jointly recommended by three major Chinese clinical guidelines as of 2023 (Chinese Society of Neurology et al., 2024; Liu et al., 2023a; Liu et al., 2023b), reflecting its recognized therapeutic value. In addition, since its approval in China in 2020, EDCSI has been widely adopted in clinical practice, and its efficacy and safety have been confirmed in the large-scale, multicenter TASTE trial (NCT02430350) (Xu et al., 2021). Furthermore, EDCSI has an established cost-effectiveness profile in previous studies (Chen et al., 2024; Shi et al., 2022; Li et al., 2024), making it a suitable benchmark. Therefore, this study compares the cost-effectiveness of EDSL versus EDCSI from the perspective of the Chinese healthcare system to provide evidence for decision-making.

## 2 Materials and methods

### 2.1 Model structure

**FIGURE 1:**
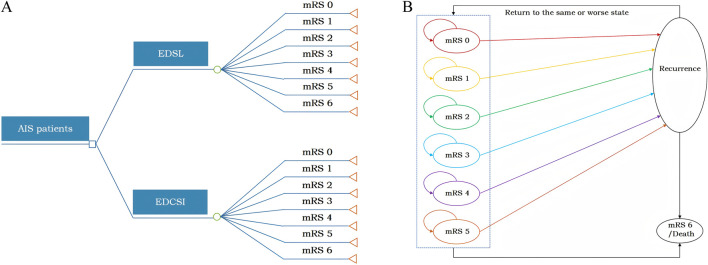
Model structure. **(A)** Short-term decision tree model; **(B)** Long-term Markov model.

This study adhered to the *Consolidated Health Economic Evaluation Reporting Standards (CHEERS 2022)* reporting guidelines (Husereau et al., 2022) and was conducted in accordance with the *China guidelines for pharmacoeconomic evaluations (2020)* (Liu et al., 2020). Based on prior studies (Chen et al., 2024; Shi et al., 2022; Li et al., 2024) and available clinical trial data (Fu et al., 2024; Xu et al., 2021), a model combining a short-term decision tree (90 days) and a long-term Markov model (lifetime) was developed in Microsoft Excel to assess the lifetime costs and health benefits of EDSL versus EDCSI in AIS patients (Figure 1).

In the short-term decision tree model (Figure 1A), patients entered with baseline modified Rankin Scale (mRS) scores 0–1 (92.2% mRS score 0, 7.8% mRS score 1) based on TASTE-SL trial data. The model included seven health states divided by mRS scores 0–6, and the distribution of mRS outcomes observed at day 90 in the clinical trial was used to calculate short-term costs and health outcomes.

The long-term Markov model (Figure 1B) included seven mRS scores health states as well as a recurrent stroke state. Based on the baseline ages of patients in the TASTE-SL and TASTE trials, the initial patient age was assumed to be 60 years old, with a cycle length of 1 year and a total time horizon of 40 years (lifetime simulation). The distribution of mRS scores at day 90 served as the initial health state distribution, and a bridging adjustment was applied during cycle 0 to extend the 90-day outcomes to the first year before entering the lifetime Markov simulation. In the model, at the end of each Markov cycle, patients may either remain in their current health state, experience a recurrent ischemic stroke and transition to a state of equal or greater disability, or die due to age-specific all-cause or excess mortality. The mRS 6 (death) state represents the final clinical outcome, wherein once entered, no further state transitions or clinical events occur.

Both costs and health outcomes were discounted at an annual rate of 5%, and a half-cycle correction was applied throughout the model (Liu et al., 2020).

### 2.2 Patients and treatments

The study targeted adult AIS patients treated within 48h of stroke onset. The intervention group received EDSL 36 mg, and the control group received EDCSI 37.5 mg via 30-min IV drip, both twice daily for 14 days.

### 2.3 Clinical data and transition probabilities

Clinical inputs for the short-term model were derived from individual patient data (IPD) from two Chinese phase III trials: TASTE-SL and TASTE. Due to differing control groups and lack of direct comparison, stabilized inverse probability of treatment weighting (sIPTW) was used to adjust for baseline differences and enhance comparability. During the weighting process, 13 key covariates were adjusted for, including sex, smoking status, drinking status, baseline National Institutes of Health Stroke Scale (NIHSS) score, history of prior stroke, stroke subtypes (large artery atherosclerosis, cardioembolic, small artery occlusion, or other etiologies), and comorbidities (history of hypertension, diabetes, hyperlipidemia, and heart disease). Both Average Treatment Effect on the Treated (ATT) and Average Treatment Effect (ATE) weighting schemes were applied: the ATT targeted the injection-treated population to assess their effects under both treatment modalities, while the ATE targeted the total population of the two groups to assess the overall effect of treatment. The base case analysis was based on the ATE weighting approach, as it better reflects real-world clinical practice where patients may receive a mix of injectable and sublingual formulations, making it more representative of the target treatment population. To ensure the accuracy of weighting estimation, patients with missing values for any baseline variables were excluded from the final analysis. After weighting, inter-group differences in proportions were assessed using chi-square tests. Balance was evaluated by calculating the standardized mean differences (SMD) for all covariates, where an absolute SMD value below 0.1 was considered indicative of negligible imbalance. Following sIPTW, the vast majority of covariates exhibited a marked reduction in SMD, falling below the 0.1 threshold. As shown in Supplementary Figures S1, S2, these results demonstrate adequate balance of baseline characteristics. Baseline characteristics before and after adjustment are presented in Supplementary Tables S1–S3.

In the efficacy evaluation, there was no statistically significant difference in the proportion of patients achieving mRS score≤1 between the EDSL and EDCSI groups (Supplementary Table S4) after sIPTW adjustment. Under ATT weighting, the proportions were 71.8% for EDSL and 67.6% for EDCSI; under ATE weighting, the proportions were 69.3% and 64.6%, respectively. Although no significant difference was observed between groups in the proportion of patients achieving mRS score≤1, AIS efficacy should also focus on the overall distributional changes of mRS scores, as functional outcomes across the 0–6 range reflect varying degrees of disability and associated health and economic consequences. Leveraging access to individual-level data, this study was able to more accurately assess how differences in the distribution of mRS 0–6 scores may influence both patient benefit and cost-effectiveness outcomes. Therefore, the complete mRS score 0–6 distribution was further analyzed. Chi-square tests indicated a statistically significant difference in the overall distribution between the two groups (Supplementary Table S5), suggesting potential differences in terms of improving various levels of functional impairment. Missing data were distributed proportionally based on the observed outcomes to maintain structural consistency. The final weighted mRS score distributions for both groups (Supplementary Table S6) were used as inputs for further evaluation.

In this study, the distribution of post-recurrence health states by mRS score (Supplementary Table S7) was based on (Gao et al., 2020). Recurrence risk, assumed uniform across mRS states, was derived from age- and sex-adjusted rates reported by (Pennlert et al., 2014). Mortality was modeled using age-specific baseline rates from *China’s Seventh National Census* (Supplementary Table S8), adjusted by mRS-specific hazard ratios (HRs) from (Hong and Saver, 2010). Parameters are detailed in Table 1.

**TABLE 1:** Model input parameters.

Parameters	Base case value	Lower value	Upper value	Distribution type	References
Hazard ratios for mortality relative to the general population					Hong and Saver (2010)
mRS 0	1.53	1.23	1.83	lognormal	
mRS 1	1.52	1.20	1.83	lognormal	
mRS 2	2.17	2.14	2.20	lognormal	
mRS 3	3.18	3.17	3.19	lognormal	
mRS 4	4.55	4.31	4.78	lognormal	
mRS 5	6.55	6.12	6.98	lognormal	
Stroke recurrent rate					Pennlert et al. (2014)
Recurrent rate (0–1 year)	5.9%	—			
Recurrent rate (1–2 years)	3.6%				
Recurrent rate (2–3 years)	2.5%				
Recurrent rate (3–4 years)	2.2%				
Recurrent rate (4–5 years)	2.2%				
Recurrent rate (5–6 years)	2.7%				
Recurrent rate (6–7 years)	2.7%				
Recurrent rate (7–8 years)	2.3%				
Recurrent rate (8–9 years)	2.8%				
Recurrent rate (9 years and above)	1.6%				
Cost (2024, Chinese Yuan)					
Edaravone Dexborneol Sublingual Tablets	70	63	77	Gamma	Menet
Edaravone Dexborneol Concentrated Injection	29.68	—	—	Fixed	National Healthcare Security Administration (2024)
Intravenous drip	8.11	7	10	Gamma	Service Price Catalogue (National Healthcare Authorities of Jiangsu, 2023)
Hospitalization costs for mRS (0–2)	12,995.74	12,804.46	13,189.61	Gamma	Pan et al. (2018)
Hospitalization costs for mRS (3–5)	17,744.26	17,355.23	18,139.75	Gamma	
Hospitalization costs for mRS (6)	14,373.51	13,207.70	15,614.28	Gamma	
Post-stroke costs for mRS (0–2)	9544.86	9248.88	9847.29	Gamma	
Post-stroke costs for mRS (3–5)	14,669.48	13,868.15	15,504.42	Gamma	
Blood urea nitrogen	6.28	4.00	7.73	Gamma	Service Price Catalogue (National Healthcare Authorities of Jiangsu, 2023)
Serum creatinine	6.51	4.00	7.73	Gamma	
Hospital days	10	—	—	Fixed	Wang et al. (2022)
Frequency of renal function monitoring (times/week)	1	0.9	1.1	Gamma	Expert Interview
Utility					Wang et al. (2025b)
mRS 0	0.983	0.981	0.985	Beta	
mRS 1	0.894	0.892	0.896	Beta	
mRS 2	0.679	0.675	0.683	Beta	
mRS 3	0.556	0.551	0.561	Beta	
mRS 4	0.270	0.263	0.277	Beta	
mRS 5	0.058	0.051	0.065	Beta	
Disutility of stroke	0.086	0.06	0.112	Beta	Mok et al. (2021)
Disutility of intravenous drip	0.023	0.0207	0.0253	Beta	Matza et al. (2013)
Others					Liu et al. (2020)
Discount rate of cost	0.05	0.00	0.08	Uniform	
Discount rate of utility	0.05	0.00	0.08		

### 2.4 Cost

From the Chinese healthcare system perspective, only direct medical costs were included, including drug, administration, and other healthcare resource costs (Table 1). Given the low adverse event rates in both groups and minimal model impact, adverse event costs and related utility decrements were excluded, consistent with prior studies (Chen et al., 2024; Shi et al., 2022; Li et al., 2024).

Drug prices were sourced from Menet and the *2024 National Insurance Catalogue* (National Healthcare Security Administration, 2024). Additional intravenous drip costs for EDCSI were averaged across five provinces (Jiangsu, Beijing, Hubei, Sichuan, Guangdong) (National Healthcare Authorities of Jiangsu, 2023). Other medical costs included renal function monitoring (weekly blood urea nitrogen and serum creatinine), hospitalization (10-day average stay (Wang et al., 2022)), and post-stroke care, with unit costs based on provincial averages, and the frequency of monitoring was determined through expert interviews. One-time hospitalization and post-stroke costs were derived from the China National Stroke Registry (CNSR) (Pan et al., 2018) and adjusted to 2024 Chinese Yuan (¥) using the medical care component of China’s Consumer Price Index.

### 2.5 Utility

The utility parameters were derived from a multicenter Chinese study of 9,978 patients using EuroQol-Five Dimension (EQ-5D) with Chinese preference weights by mRS scores (Wang L. et al., 2025). The model included disutilities from both disease and treatment: AIS disutility referenced cerebrovascular burden in type 2 diabetes patients from (Mok et al., 2021), while a one-time disutility for 30-min intravenous drip was based on treatment preference data from (Matza et al., 2013). Utility parameters are shown in Table 1.

### 2.6 Cost-effectiveness analysis

Cost-effectiveness was assessed using the incremental cost-effectiveness ratio (ICER), with a willingness-to-pay (WTP) threshold set at 1–3 times the 2024 gross domestic product (GDP) *per capita* (¥95,749) (National Bureau of Statistics of China, 2024), in line with *China guidelines for pharmacoeconomic evaluations (2020)* (Liu et al., 2020). Treatments with ICERs <¥95,749 were considered highly cost-effective, ¥95,749–287,247 as cost-effective, and >¥287,247 as not cost-effective.

### 2.7 Sensitivity analyses

Deterministic sensitivity analysis (DSA), probabilistic sensitivity analysis (PSA), and scenario analysis were performed to test the robustness of base-case results. In one-way DSA, individual parameters were varied within defined ranges, and impacts on ICERs were visualized using a tornado diagram. PSA with 5,000 Monte Carlo iterations generated a cost-effectiveness acceptability curve (CEAC) to estimate the probability of EDSL being cost-effective versus EDCSI. In addition, considering the long time horizon of the study, scenario analyses explored alternative time horizons (90 days, 5, 10, 20, and 30 years) to assess their influence on model outcomes. The ATT-based results were included in the scenario analyses to provide supplementary validation of the base-case findings.

## 3 Results

### 3.1 Base case analysis

EDSL demonstrated absolute cost-effectiveness advantages over EDCSI under the ATE-weighted populations (Table 2). The ICERs showed cost savings of ¥2,089.84 per QALY gained.

**TABLE 2:** Base-case costs and health outcomes results.

Results	EDSL	EDCSI	Increment
Costs	¥27,892.83	¥28,976.34	-¥1,083.51
Drug costs	¥1,960.00	¥2,493.12	-¥533.12
Administration costs	¥0.00	¥226.97	-¥226.97
Renal function monitoring costs	¥25.57	¥25.57	¥0.00
Hospitalization costs	¥18,179.12	¥18,389.12	-¥210.00
Post-stroke costs	¥7,728.14	¥7,841.56	-¥113.42
QALYs	8.61	8.09	0.52
Health status utility	8.67	8.18	0.50
Disutility	−0.07	−0.09	0.02
ICER	-¥2,089.84		
Result	More Effective & Less Costly		

### 3.2 One-way sensitivity analysis

**FIGURE 2:**
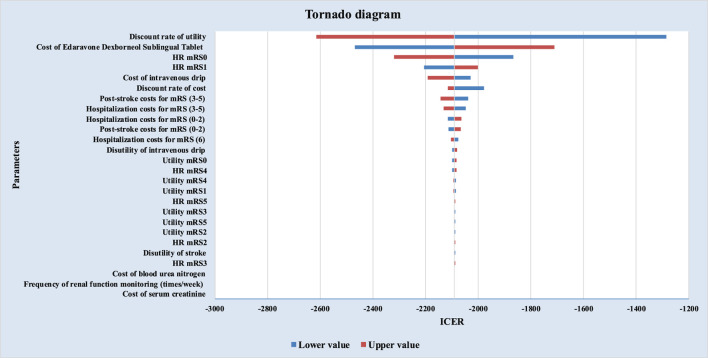
Tornado diagram. under ATE weighting.

As shown in Figure 2, under ATE-weighted population distribution, the three most influential parameters in the one-way sensitivity analysis were: the discount rate for utility, the unit price of EDSL, the mortality HR for mRS 0 patients. EDSL consistently demonstrated absolute dominance over EDCSI for the treatment of AIS, in line with the base-case results.

### 3.3 Probabilistic sensitivity analysis

**FIGURE 3:**
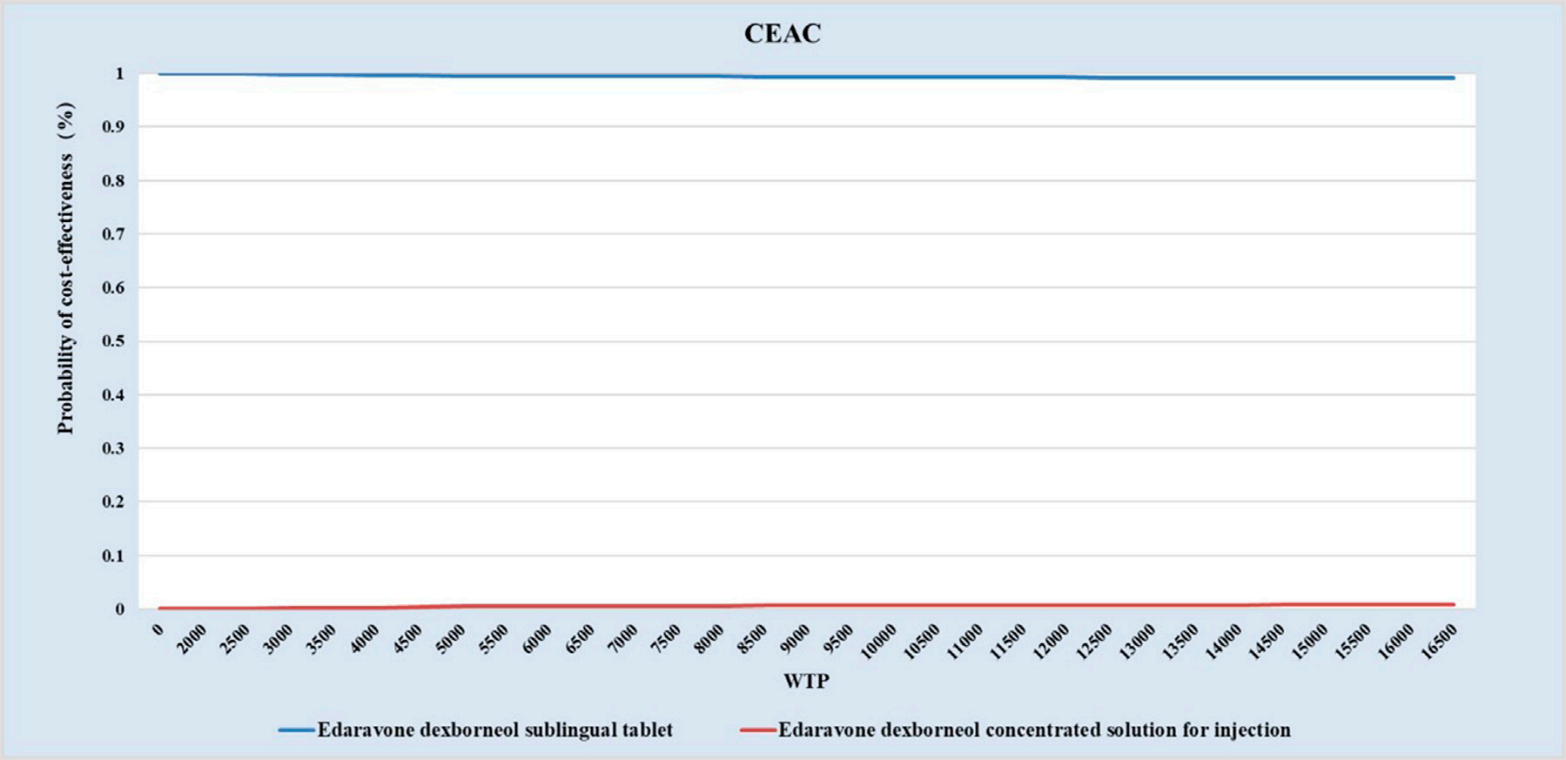
Probabilistic sensitivity analysis results:CEAC generated under ATE weighting.

**FIGURE 4:**
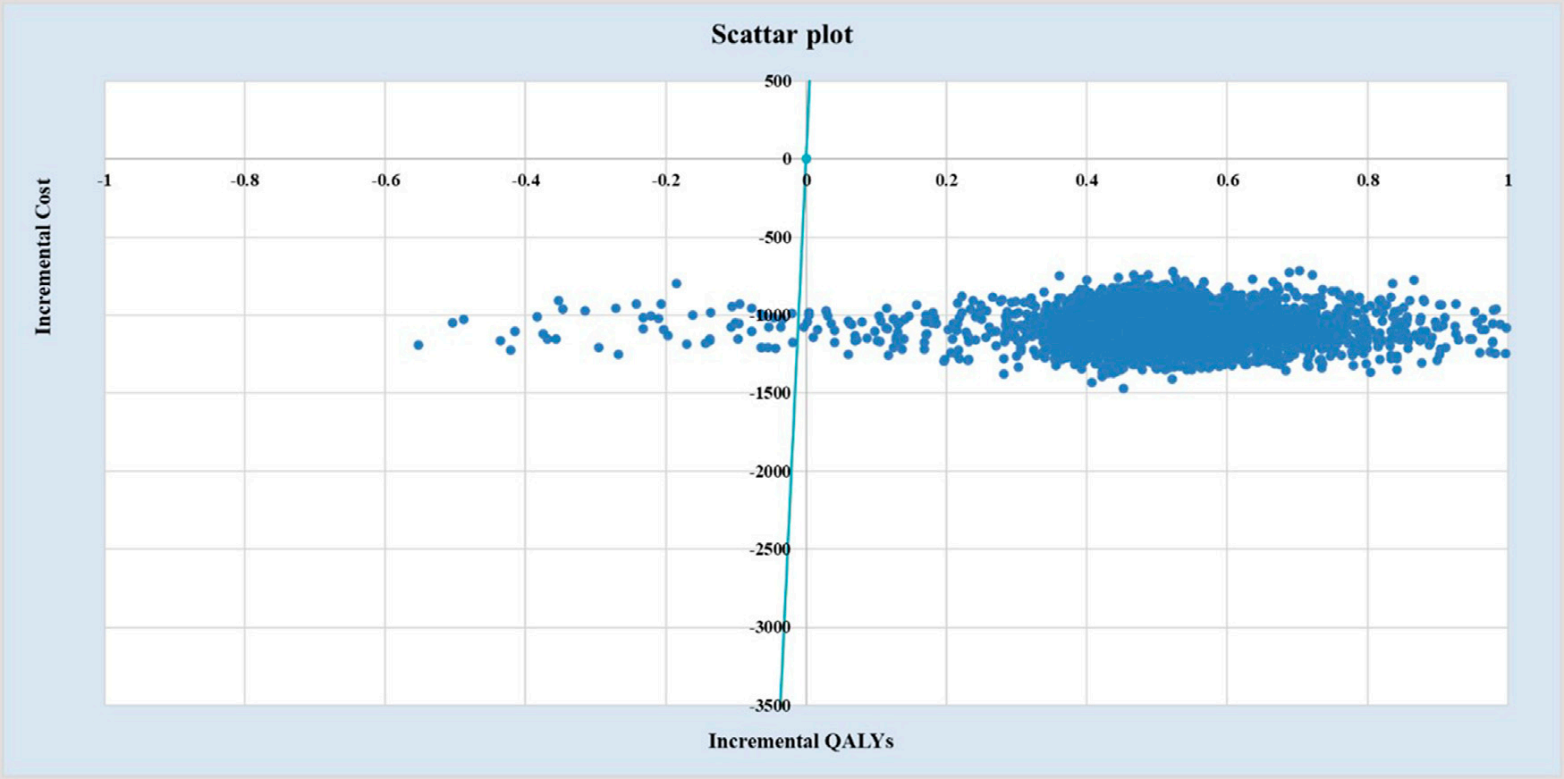
Probabilistic sensitivity analysis results:ICER scatterplot under ATE weighting.

As shown in Figures 3, 4, under ATE-weighted population distribution, the probability that EDSL is cost-effective remained close to 100% across all WTP thresholds.

### 3.4 Scenario analysis

As shown in Table 3, EDSL maintained absolute advantages over EDCSI across all time horizons (90 days–30 years) under ATE-weighted analyses, reinforcing the robustness of the base-case results.

**TABLE 3:** Scenario analysis results based on alternative time horizons.

Results	90 days	5 years	10 years	20 years	30 years
Incremental costs (EDSL-EDCSI)	-¥1,043.92	-¥1,117.79	-¥1,119.22	-¥1,099.31	-¥1,085.12
Incremental QALYs(EDSL-EDCSI)	0.03	0.20	0.33	0.48	0.52
ICER	-¥36,749.24	-¥5,514.15	-¥3,371.84	-¥2,313.78	-¥2,105.39
Results	All are “More Effective & Less Costly”				

In the ATT-weighted scenario analysis, each additional QALY gained yielded a cost saving of ¥2,260.24 (Supplementary Table S9), confirming the cost-effectiveness advantage of EDSL over EDCSI. DSA and PSA were also performed under ATT weighting, and the results, presented in Supplementary Figures S3–5, demonstrated consistent trends, further supporting the robustness of the findings. Moreover, the impact of varying the time horizon was assessed, and under all tested durations (90 days–30 years), EDSL consistently showed absolute cost-effectiveness advantages over EDCSI in the ATT-weighted population (Supplementary Table S10). These findings provide additional confirmation of the stability and reliability of the base-case results.

## 4 Discussion

From the perspective of the Chinese healthcare system, this study used a combined short-term decision tree and long-term Markov model to evaluate the cost-effectiveness of EDSL versus EDCSI for AIS treatment. The analysis showed that EDSL achieved comparable clinical efficacy while incurring lower overall treatment costs. The ICER was –¥2,089.84 per QALY gained, indicating that EDSL is a cost-saving option. Sensitivity analyses confirmed the robustness of these findings across a range of model parameters and assumptions.

For indirect treatment comparison, this study applied sIPTW based on data from the TASTE-SL (Fu et al., 2024) and TASTE trials (Xu et al., 2021), adjusting for key baseline covariates under both ATT and ATE frameworks to enhance robustness and generalizability. While ATE was used as the base-case analysis to reflect the broader real-world population, an additional analysis using ATT was conducted. The consistency of results across both weighting methods further supports the reliability of the findings. Despite differences in trial year and sample size, the two studies shared highly consistent designs, criteria, and outcomes, supporting the methodological validity of sIPTW. Moreover, the model incorporated large-scale real-world utility data from a Chinese population and accounted for utility changes associated with both treatment and clinical events (Wang L. et al., 2025; Mok et al., 2021; Matza et al., 2013), thereby improving its representativeness. Additionally, the combined short-term decision tree and long-term Markov model used in this study has been widely applied in economic evaluations related to stroke (Chen et al., 2024; Shi et al., 2022; Li et al., 2024), which captured long-term health transitions and cost-effectiveness outcomes following AIS treatment, offering both strong generalizability and explanatory power.

Previous studies have demonstrated that edaravone dexborneol concentrated solution for injection (EDCSI) offers superior cost-effectiveness in the treatment of AIS compared with other cytoprotective therapies such as DI-3-n-butylphthalide, human urinary kallidinogenase, and so on (Chen et al., 2024; Shi et al., 2022; Li et al., 2024). Building upon these findings, the current study showed that EDSL further reduced treatment costs while gaining more QALYs. This not only continues the economic advantages of edaravone dexborneol but also enhances medication convenience and patient adherence through formulation innovation, indicating its potential as a more favorable treatment option for AIS. Moreover, the findings of this study are highly consistent with those of a recent systematic review and meta-analysis. Kashbour et al. (2025) conducted a meta-analysis of five randomized controlled trials (involving 2,535 patients). The results demonstrated that edaravone (ED) treatment significantly increased the proportion of patients achieving functional independence (mRS ≤1) at 90 days in acute ischemic stroke patients. Additionally, the analysis revealed a significant improvement in NIHSS scores at both 30 and 90 days. These findings demonstrate the efficacy and safety advantages of ED-class drugs. Future research could focus on direct economic comparisons between the sublingual tablet and other cytoprotective therapies to inform clinical and reimbursement decisions.

There are also limitations to this study. First, this study is based on two randomized controlled trials (TASTE-SL and TASTE) that enrolled Chinese patients only. All model parameters—including clinical outcomes, costs, and utility values—were derived from China-specific data. While this approach enhances the internal validity of the results within the Chinese healthcare system, it may also limit their direct applicability to other countries or healthcare settings. When applying the findings of this study, adaptation based on local factors such as drug pricing, medical resource costs, clinical practice patterns, and willingness-to-pay thresholds is recommended. Second, some key model parameters, such as stroke recurrence rates and hazard ratios for mortality relative to the general population, were derived from non-Chinese studies (Pennlert et al., 2014; Hong and Saver, 2010), which may affect the estimate precision due to population differences. However, sensitivity analyses ensured the robustness of the results. This limitation is acknowledged explicitly, and it is recommended that future studies utilize long-term follow-up data from Chinese populations, along with uncertainty information, for further validation. Nevertheless, by employing transparent, widely accepted data sources and maintaining consistency in parameter structure, this study provides valuable decision-making guidance within the current evidence landscape. In addition, as the analysis adopted a healthcare system perspective, indirect costs (e.g., productivity loss of patients and caregivers) were excluded, potentially underestimating the broader economic value of the sublingual tablet, particularly in improving adherence and reducing caregiver burden.

Importantly, functional impairments resulting from AIS are often severe and persistent: 70%–80% of AIS patients experience motor impairments; 80% have early-stage limb weakness; 70% have speech difficulties; 73% experience visual problems; and over half develop cognitive decline within 6 months (Lu et al., 2021; Rowe et al., 2019). These persistent deficits not only affect patient recovery but also significantly increase the burden on caregivers. Studies have shown that 96.5% report moderate to severe caregiving burden, over half experience symptoms of anxiety or depression, and 80% consider the financial burden to be overwhelming (Lv et al., 2016; Das et al., 2010). Given this dual impact on patients and caregivers, neuroprotection has become a critical focus in AIS management. The Stroke Therapy Academic Industry Roundtable (STAIR) and the Stroke Preclinical Assessment Network (SPAN) have endorsed brain cytoprotective therapies as an important component of AIS management (Albers et al., 2011; Lyden et al., 2022). Similarly, Chinese clinical guidelines recommend several neuroprotective agents for AIS treatment (Wang et al., 2023; Ni et al., 2021; Geng et al., 2022), with edaravone dexborneol being one of the most prominently supported options due to its dual mechanisms of free radical scavenging and anti-inflammatory activity, as well as its well-established efficacy and safety profile. Both the injectable formulation and the sublingual tablet have been introduced into clinical use, offering greater flexibility in treatment planning. In clinical settings, EDCSI and EDSL may serve complementary roles within a full-course, sequential treatment strategy. Patients are often treated with the injectable form during hospitalization and transitioned to the sublingual tablet upon discharge. Under such a regimen, healthcare costs may reflect combined use rather than a simple one-to-one substitution. This approach may help maintain therapeutic plasma concentrations and ensure continuity of treatment. However, the equivalence of therapeutic outcomes between formulations, particularly in sequential use, warrants further validation through real-world evidence. Chinese clinical guidelines also emphasize the role of neuroprotection in AIS management and recommend multiple cytoprotective therapies such as edaravone dexborneol, DI-3-n-butylphthalide, human urinary kallidinogenase, and vinpocetine (Chinese Society of Neurology et al., 2024; Liu et al., 2023a) (Liu et al., 2023b), which have demonstrated their efficacy in improving neurological function and reducing post-stroke sequelae.

In summary, EDSL combines economic, clinical, and practical advantages through formulation innovation, supporting its broader adoption in the treatment of AIS.

## 5 Conclusion

This study demonstrates that, from the perspective of the Chinese healthcare system, EDSL is more cost-effective than EDCSI for treating AIS. Its convenient sublingual administration also enhances treatment continuity and accessibility in real-world settings.

## Data Availability

The original contributions presented in the study are included in the article/Supplementary Material, further inquiries can be directed to the corresponding author.

## References

[B1] Albers G. W. Goldstein L. B. Hess D. C. Wechsler L. R. Furie K. L. Gorelick P. B. (2011). Stroke treatment academic industry roundtable (STAIR) recommendations for maximizing the use of intravenous thrombolytics and expanding treatment options with intra-arterial and neuroprotective therapies. Stroke 42 (9), 2645–2650. 10.1161/STROKEAHA.111.618850 21852620

[B2] Chen P. Luo M. Chen Y. Zhang Y. Wang C. Li H. (2024). Cost-effectiveness of edaravone dexborneol *versus* human urinary kallidinogenase for acute ischemic stroke in China. Health Econ. Rev. 14 (1), 7. 10.1186/s13561-024-00479-6 38285185 PMC10823610

[B3] Chinese Society of Neurology, Chinese Stroke Society (2024). Chinese guidelines for diagnosis and treatment of acute ischemic stroke 2023. Chin. J. Neurology 57 (6), 523–559. 10.3760/cma.j.cn113694-20240410-00221

[B4] Das S. Hazra A. Ray B. K. Ghosal M. Banerjee T. K. Roy T. (2010). Burden among stroke caregivers: results of a community-based study from kolkata, India. Stroke 41 (12), 2965–2968. 10.1161/STROKEAHA.110.589598 20947851

[B5] Fu Y. Wang A. Tang R. Tian X. Xia X. (2024). Sublingual edaravone dexborneol for the treatment of acute ischemic stroke: the TASTE-SL randomized clinical trial. JAMA Neurol. 81 (4), 319–326. 10.1001/jamaneurol.2023.5716 38372981 PMC10877503

[B6] Gao L. Moodie M. Mitchell P. J. Churilov L. Kleinig T. J. Yassi N. (2020). Cost-Effectiveness of tenecteplase before thrombectomy for ischemic stroke. Stroke 51 (12), 3681–3689. 10.1161/STROKEAHA.120.029666 33023423

[B7] GBD 2019 Stroke Collaborators (2021). Global, regional, and national burden of stroke and its risk factors, 1990-2019: a systematic analysis for the global burden of disease study 2019. Lancet Neurol. 20 (10), 795–820. 10.1016/S1474-4422(21)00252-0 34487721 PMC8443449

[B8] Geng B. Zhao X. Song Z. (2022). Effect of bufenopeptide combined with vinpocetine on serum levels of lncRNA CAI2 and CD62P in patients with acute ischemic stroke. Hebei Med. J. 44 (24), 3721–3724+3729. 10.3969/j.issn.1002-7386.2022.24.008

[B9] Hong K. S. Saver J. L. (2010). Years of disability-adjusted life gained as a result of thrombolytic therapy for acute ischemic stroke. Stroke 41 (3), 471–477. 10.1161/STROKEAHA.109.571083 20133917

[B10] Husereau D. Drummond M. Augustovski F. de Bekker-Grob E. Briggs A. H. Carswell C. (2022). Consolidated health economic evaluation reporting standards 2022 (CHEERS 2022) statement: updated reporting guidance for health economic evaluations. Value Health 25 (1), 3–9. 10.1016/j.jval.2021.11.1351 35031096

[B11] Kashbour M. Shata A. Wagdy M. Alnajjar A. Z. Aldemerdash M. A. Tarakhan H. (2025). Efficacy and safety of edaravone dexborneol in acute ischemic stroke: systematic review and meta-analysis of randomized controlled trials. Naunyn Schmiedeb. Arch. Pharmacol. 398 (8), 9569–9582. 10.1007/s00210-025-03950-1 40047860

[B12] Li J. Cao W. Zhao F. Jin P. (2024). Cost-effectiveness of edaravone dexborneol *versus* dl-3-n-butylphthalide for the treatment of acute ischemic stroke: a Chinese health care perspective. BMC Public Health 24 (1), 436. 10.1186/s12889-024-17959-3 38347500 PMC10860239

[B13] Liu G. Hu S. Wu J. (2020). China guidelines for pharmacoeconomic evaluations(2020). Beijing: China Market Press.

[B14] Liu L. Zhou H. Duan W. (2023a). Chinese stroke association guidelines for clinical management of cerebrovascular diseases (second edition) (excerpt)——chapter four clinical management of ischaemic cerebrovascular diseases. Chin. J. Stroke 18 (8), 910–933. 10.3969/j.issn.1673-5765.2023.08.009

[B15] Liu L. Li Z. Zhou H. Duan W. Huo X. Xu W. (2023b). Chinese Stroke Association guidelines for clinical management of ischaemic cerebrovascular diseases: executive summary and 2023 update. Stroke Vasc. Neurol. 8 (6), e3. 10.1136/svn-2023-002998 38158224 PMC10800268

[B16] Lu X. Niu X. Shen C. Liu F. Liu Z. Huang K. (2021). Development and validation of a polygenic risk Score for stroke in the Chinese population. Neurology 97 (6), e619–e628. 10.1212/WNL.0000000000012263 34031205 PMC8424497

[B17] Lv L. Guo H. Hu L. (2016). Correlation between self-efficacy and care ability of stroke patients' family caregivers. Mod. Clin. Nurs. 15 (08), 6–10. 10.3969/j.issn.1671-8283.2016.08.002

[B18] Lyden P. D. Bosetti F. Diniz M. A. Rogatko A. Koenig J. I. Lamb J. (2022). The stroke preclinical assessment network: rationale, design, feasibility, and stage 1 results. Stroke 53 (5), 1802–1812. 10.1161/STROKEAHA.121.038047 35354299 PMC9038686

[B19] Matza L. S. Cong Z. Chung K. Stopeck A. Tonkin K. Brown J. (2013). Utilities associated with subcutaneous injections and intravenous infusions for treatment of patients with bone metastases. Patient Prefer Adherence 7, 855–865. 10.2147/PPA.S44947 24039408 PMC3770342

[B20] Mok C. H. Kwok H. H. Y. Ng C. S. Leung G. M. Quan J. (2021). Health State utility values for type 2 diabetes and related complications in east and southeast Asia: a systematic review and meta-analysis. Value Health 24 (7), 1059–1067. 10.1016/j.jval.2020.12.019 34243830

[B21] National Bureau of Statistics of China (2024). Statistical bulletin on national economic and social development of the People'S Republic of China. Available online at: https://www.stats.gov.cn/sj/zxfb/202502/t20250228_1958817.html (Accessed April 15, 2025).

[B22] National Healthcare Authorities of Jiangsu (2023). Sichuan, and Guangdong. Service price catalogue of five provinces and municipalities.

[B23] National Healthcare Security Administration (2024). Ministry of human resources and social security. Notice on the publication of the national drug reimbursement list for basic medical insurance, work injury insurance, and maternity insurance. Available online at: https://www.gov.cn/zhengce/zhengceku/202411/content_6989859.htm (Accessed April 15, 2025).

[B24] Ni J. Yao M. Wang L. H. Yu M. Li R. H. Zhao L. H. (2021). Human urinary kallidinogenase in acute ischemic stroke: a single-arm, multicenter, phase IV study (RESK study). CNS Neurosci. Ther. 27 (12), 1493–1503. 10.1111/cns.13724 34510762 PMC8611767

[B25] Pan Y. Cai X. Huo X. Zhao X. Liu L. Wang Y. (2018). Cost-effectiveness of mechanical thrombectomy within 6 hours of acute ischaemic stroke in China. BMJ Open 8 (2), e018951. 10.1136/bmjopen-2017-018951 29472264 PMC5855394

[B26] Peng C. Chen J. Li J. (2022). Study of the economic burden and infuencing factors on stroke patients in Central China in the post-epidemic era. J. Chin. Pharm. Sci. 31 (07), 545–555. 10.5246/jcps.2022.07.048

[B27] Pennlert J. Eriksson M. Carlberg B. Wiklund P. G. (2014). Long-term risk and predictors of recurrent stroke beyond the acute phase. Stroke 45 (6), 1839–1841. 10.1161/STROKEAHA.114.005060 24788972

[B28] Rowe F. J. Hepworth L. R. Howard C. Hanna K. L. Cheyne C. P. Currie J. (2019). High incidence and prevalence of visual problems after acute stroke: an epidemiology study with implications for service delivery. PLoS One 14 (3), e0213035. 10.1371/journal.pone.0213035 30840662 PMC6402759

[B29] Shi F. He Z. Wang L. Su H. Han S. (2022). Cost-effectiveness of edaravone dexborneol *versus* edaravone for the treatment of acute ischemic stroke in China: based on the TASTE study. Front. Pharmacol. 13, 938239. 10.3389/fphar.2022.938239 36330098 PMC9622952

[B30] Wang Y. Li Z. Gu H. (2022). China stroke statistics 2020. Chin. J. Stroke 17 (5), 433–447. 10.3969/j.issn.1673-5765.2022.05.001

[B31] Wang A. Jia B. Zhang X. Huo X. Chen J. Gui L. (2023). Efficacy and safety of butylphthalide in patients with acute ischemic stroke: a randomized clinical trial. JAMA Neurol. 80 (8), 851–859. 10.1001/jamaneurol.2023.1871 37358859 PMC10294018

[B32] Wang Y. Zhang Y. Y. Yan J. Ji T. B. Fan L. D. Wang H. d. (2025a). The impact of diagnosis-related group-based medical insurance payment model on the prognosis and nursing care of patients undergoing composite trabeculectomy: a retrospective cohort study. Front. Public Health 13, 1518546. 10.3389/fpubh.2025.1518546 40469599 PMC12133863

[B33] Wang L. Guan X. Zhou J. Hu H. Liu W. Wei Q. (2025b). Measuring the health outcomes of Chinese ischemic stroke patients based on the data from a longitudinal multi-center study. Qual. Life Res. 34, 1967–1977. 10.1007/s11136-025-03957-4 40146503 PMC12182522

[B34] Xie D. Zhang Y. Zhu G. (2024). Ischemic stroke: mechanisms to treatment (Review). Adv. Clin. Med. 14 (6), 838–853. 10.12677/acm.2024.1461850

[B35] Xu J. Wang A. Meng X. Yalkun G. Xu A. Gao Z. (2021). Edaravone dexborneol *versus* edaravone alone for the treatment of acute ischemic stroke: a phase III, randomized, double-blind, comparative trial. Stroke 52 (3), 772–780. 10.1161/STROKEAHA.120.031197 33588596

[B36] Yuan J. Lu Z. K. Xiong X. Li M. Liu Y. Wang L. D. (2023). Age and geographic disparities in acute ischaemic stroke prehospital delays in China: a cross-sectional study using national stroke registry data. Lancet Reg. Health West Pac 33, 100693. 10.1016/j.lanwpc.2023.100693 37181525 PMC10166992

