# Supplementary material for: Cost-effectiveness of edaravone dexborneol sublingual tablet versus concentrated solution for injection for the treatment of acute ischemic stroke in China
Source: Front Pharmacol. 2025 Nov 13;16:1661581. doi: 10.3389/fphar.2025.1661581 (PMC12658356; doi:10.3389/fphar.2025.1661581)
Supplement: Supplementary file 1 [file Supplementaryfile1.docx]

**Appendix**

**Supplementary Table S1** Baseline Characteristics of TASTE-SL and TASTE Patients Before sIPTW Processing.

**Supplementary Table S2** Baseline Characteristics of TASTE-SL and TASTE Patients After ATT Weighting.

**Supplementary Table S3** Baseline Characteristics of TASTE-SL and TASTE Patients After ATE Weighting.

**Supplementary Table S4** Comparison of mRS score≤1 between EDSL and EDCI after sIPTW processing.

**Supplementary Table S5** Distribution of all mRS scores at D90 for EDSL and EDCSI before and after sIPTW adjustment.

**Supplementary Table S6** Distribution of all mRS scores at D90 for edaravone dexborneol sublingual tablets and concentrated injection as used in the model.

**Supplementary Table S7** Distribution after recurrent stroke.

**Supplementary Table S8** China Life Table.

**Supplementary Table S9** Base-case costs and health outcomes under ATT weighting.

**Supplementary Table S10** Cost-effectiveness outcomes across alternative time horizons under ATT weighting.

**Supplementary Figure S1** Tornado diagram under ATT weighting.

**Supplementary Figure S2** Probabilistic sensitivity analysis results. A CEAC generated under ATT weighting, B ICER scatterplot under ATT weighting.

**Supplementary Table S1** Baseline Characteristics of TASTE-SL and TASTE Patients Before sIPTW Processing.

|  | **EDSL(N/%)** | **EDCSI(N/%)** | **P-value** |
| --- | --- | --- | --- |
| **Sex** | | | |
| **Male** | 292(69.03) | 389(67.07) | 0.4998 |
| **Female** | 131(30.97) | 191(32.93) |  |
| **Smoke** | | | |
| **No** | 317(74.94) | 357(61.55) | **0.0000** |
| **Yes** | 106(25.06) | 223(38.45) |  |
| **Drink** | | | |
| **No** | 408(96.45) | 515(88.79) | **0.0000** |
| **Yes** | 15(3.55) | 65(11.21) |  |
| **NIHSS<8** | | | |
| **No** | 169(39.95) | 198(34.14) | 0.0539 |
| **Yes** | 254(60.05) | 382(65.86) |  |
| **History of Prior Stroke** | | | |
| **No** | 306(72.34) | 413(71.21) | 0.6871 |
| **Yes** | 117(27.66) | 167(28.79) |  |
| **Large Artery Atherosclerotic Type** | | | |
| **No** | 192(45.39) | 241(41.55) | 0.2143 |
| **Yes** | 231(54.61) | 339(58.45) |  |
| **Cardioembolic Type** | | | |
| **No** | 414(97.87) | 549(94.66) | **0.0050** |
| **Yes** | 9(2.13) | 31(5.34) |  |
| **Small Artery Occlusion Type** | | | |
| **No** | 248(58.63) | 394(67.93) | **0.0020** |
| **Yes** | 175(41.37) | 186(32.07) |  |
| **Other Etiologies** | | | |
| **No** | 422(99.76) | 577(99.48) | 0.4536 |
| **Yes** | 1(0.24) | 3(0.52) |  |
| **History of Hypertension** | | | |
| **No** | 75(17.73) | 162(27.93) | **0.0001** |
| **Yes** | 348(82.27) | 418(72.07) |  |
| **History of Diabetes** | | | |
| **No** | 251(59.34) | 409(70.52) | **0.0002** |
| **Yes** | 172(40.66) | 171(29.48) |  |
| **History of Hyperlipidemia** | | | |
| **No** | 219(51.77) | 365(62.93) | **0.0003** |
| **Yes** | 204(48.23) | 215(37.07) |  |
| **History of Heart Disease** | | | |
| **No** | 301(71.16) | 448(77.24) | **0.0264** |
| **Yes** | 122(28.84) | 132(22.76) |  |

**Supplementary Table S2** Baseline Characteristics of TASTE-SL and TASTE Patients After ATT Weighting.

|  | **EDSL(N/%)** | **EDCSI(N/%)** | **P-value** |
| --- | --- | --- | --- |
| **Sex** | | | |
| **Male** | 276(65.22) | 389(67.07) | 0.5401 |
| **Female** | 147(34.78) | 191(32.93) |  |
| **Smoke** | | | |
| **No** | 269(63.54) | 357(61.55) | 0.5219 |
| **Yes** | 154(36.47) | 223(38.45) |  |
| **Drink** | | | |
| **No** | 384(90.76) | 515(88.79) | 0.3151 |
| **Yes** | 39(9.25) | 65(11.21) |  |
| **NIHSS<8** | | | |
| **No** | 134(31.58) | 198(34.14) | 0.3948 |
| **Yes** | 289(68.42) | 382(65.86) |  |
| **History of Prior Stroke** | | | |
| **No** | 282(66.71) | 413(71.21) | 0.1272 |
| **Yes** | 141(33.29) | 167(28.79) |  |
| **History of Prior Stroke** | | | |
| **No** | 181(42.9) | 241(41.55) | 0.6709 |
| **Yes** | 242(57.11) | 339(58.45) |  |
| **Cardioembolic Type** | | | |
| **No** | 392(92.59) | 549(94.66) | 0.1799 |
| **Yes** | 31(7.42) | 31(5.34) |  |
| **Small Artery Occlusion Type** | | | |
| **No** | 292(68.93) | 394(67.93) | 0.7379 |
| **Yes** | 131(31.08) | 186(32.07) |  |
| **Other Etiologies** | | | |
| **No** | 421(99.64) | 577(99.48) | 0.7180 |
| **Yes** | 2(0.36) | 3(0.52) |  |
| **History of Hypertension** | | | |
| **No** | 115(27.27) | 162(27.93) | 0.8160 |
| **Yes** | 308(72.74) | 418(72.07) |  |
| **History of Diabetes** | | | |
| **No** | 304(71.8) | 409(70.52) | 0.6586 |
| **Yes** | 119(28.2) | 171(29.48) |  |
| **History of Hyperlipidemia** | | | |
| **No** | 272(64.36) | 365(62.93) | 0.6586 |
| **Yes** | 151(35.64) | 215(37.07) |  |
| **History of Heart Disease** | | | |
| **No** | 337(79.62) | 448(77.24) | 0.3687 |
| **Yes** | 86(20.39) | 132(22.76) |  |

**Supplementary Table S3** Baseline Characteristics of TASTE-SL and TASTE Patients After ATE Weighting.

|  | **EDSL(N/%)** | **EDCSI(N/%)** | **P-value** |
| --- | --- | --- | --- |
| **Sex** | | | |
| **Male** | 283(66.83) | 397(68.41) | 0.5951 |
| **Female** | 140(33.17) | 184(31.59) |  |
| **Smoke** | | | |
| **No** | 289(68.35) | 390(67.18) | 0.6962 |
| **Yes** | 134(31.65) | 191(32.82) |  |
| **Drink** | | | |
| **No** | 394(93.16) | 535(92.14) | 0.5442 |
| **Yes** | 29(6.84) | 46(7.86) |  |
| **NIHSS<8** | | | |
| **No** | 169(39.95) | 198(34.14) | 0.1270 |
| **Yes** | 254(60.05) | 382(65.86) |  |
| **History of Prior Stroke** | | | |
| **No** | 292(69.09) | 411(70.79) | 0.5598 |
| **Yes** | 131(30.92) | 170(29.21) |  |
| **History of Prior Stroke** | | | |
| **No** | 186(43.95) | 251(43.15) | 0.8017 |
| **Yes** | 237(56.06) | 330(56.85) |  |
| **Cardioembolic Type** | | | |
| **No** | 401(94.82) | 558(95.97) | 0.3826 |
| **Yes** | 22(5.19) | 23(4.03) |  |
| **Small Artery Occlusion Type** | | | |
| **No** | 273(64.59) | 373(64.21) | 0.9021 |
| **Yes** | 150(35.42) | 208(35.79) |  |
| **Other Etiologies** | | | |
| **No** | 422(99.69) | 579(99.63) | 0.8748 |
| **Yes** | 1(0.31) | 2(0.37) |  |
| **History of Hypertension** | | | |
| **No** | 98(23.25) | 136(23.37) | 0.9625 |
| **Yes** | 325(76.76) | 445(76.63) |  |
| **History of Diabetes** | | | |
| **No** | 281(66.55) | 388(66.77) | 0.9400 |
| **Yes** | 142(33.46) | 193(33.23) |  |
| **History of Hyperlipidemia** | | | |
| **No** | 250(59.05) | 341(58.72) | 0.9154 |
| **Yes** | 173(40.95) | 240(41.28) |  |
| **History of Heart Disease** | | | |
| **No** | 322(76.05) | 438(75.32) | 0.7902 |
| **Yes** | 101(23.95) | 143(24.68) |  |

**Supplementary Table S4** Comparison of mRS score≤1 between EDSL and EDCI after sIPTW processing.

|  | **EDSL**  **(mRS≤1)** | **EDCSI**  **(mRS≤1)** | **Difference (95% CI) (EDSL-EDCSI)** | **P-value** |
| --- | --- | --- | --- | --- |
| **ATT** | 71.8% | 67.6% | 4.2%（-1.5%, 10.0%） | **0.1505** |
| **ATE** | 69.3% | 64.6% | 4.6%（-1.2%, 10.5%） | **0.1246** |

**Supplementary Table S5** Distribution of all mRS scores at D90 for EDSL and EDCSI before and after sIPTW adjustment.

| **Distribution of mRS scores at D90** | Unweighted | | ATT | | ATE | |
| --- | --- | --- | --- | --- | --- | --- |
|  | EDSL | EDCSI | EDSL | EDCSI | EDSL | EDCSI |
| 0 | 27.78% | 22.2% | 33.48% | 22.76% | 31.63% | 20.72% |
| 1 | 36.67% | 43.41% | 38.34% | 44.83% | 37.62% | 43.9% |
| 2 | 11.56% | 12.52% | 10.71% | 12.93% | 11.08% | 11.94% |
| 3 | 12.44% | 10.68% | 10.06% | 11.03% | 11.2% | 12.79% |
| 4 | 6.22% | 6.18% | 5.15% | 6.38% | 5.57% | 8.03% |
| 5 | 0.89% | 1.34% | 0.73% | 1.38% | 0.82% | 1.65% |
| 6 | 0.44% | 1.34% | 0.16% | 0.69% | 0.29% | 0.98% |
| Missing value | 4% | 2.34% | 1.37% | 0% | 1.79% | 0% |
| P-value | —— | | **0.0008*** | | **0.0033*** | |

**Supplementary Table S6** Distribution of all mRS scores at D90 for edaravone dexborneol sublingual tablets and concentrated injection as used in the model.

| **Distribution of mRS scores at D90** | **ATT** | | **ATE** | |
| --- | --- | --- | --- | --- |
|  | **EDSL** | **EDCSI** | **EDSL** | **EDCSI** |
| 0 | 33.95% | 22.76% | 32.20% | 20.72% |
| 1 | 38.87% | 44.83% | 38.31% | 43.90% |
| 2 | 10.86% | 12.93% | 11.28% | 11.94% |
| 3 | 10.20% | 11.03% | 11.41% | 12.79% |
| 4 | 5.22% | 6.38% | 5.67% | 8.03% |
| 5 | 0.74% | 1.38% | 0.84% | 1.65% |
| 6 | 0.16% | 0.69% | 0.30% | 0.98% |

**Supplementary Table S7** Distribution after recurrent stroke.

| **Post-recurrent mRS state** | **mRS state before recurrent** | | | | | |
| --- | --- | --- | --- | --- | --- | --- |
|  | **mRS 0** | **mRS 1** | **mRS 2** | **mRS 3** | **mRS 4** | **mRS 5** |
| **mRS 0** | 17.82% |  |  |  |  |  |
| **mRS 1** | 22.77% | 40.59% |  |  |  |  |
| **mRS 2** | 8.91% | 8.91% | 49.50% |  |  |  |
| **mRS 3** | 11.88% | 11.88% | 11.88% | 61.38% |  |  |
| **mRS 4** | 13.86% | 13.86% | 13.86% | 13.86% | 75.24% |  |
| **mRS 5** | 6.93% | 6.93% | 6.93% | 6.93% | 6.93% | 82.17% |
| **mRS 6** | 17.83% | 17.83% | 17.83% | 17.83% | 17.83% | 17.83% |
| **Sum** | **100.00%** | **100.00%** | **100.00%** | **100.00%** | **100.00%** | **100.00%** |

**Supplementary Table S8** China Life Table.

| Age | Natural mortality rate, per year |
| --- | --- |
| 0 | 0.1530% |
| 1 | 0.0390% |
| 2 | 0.0260% |
| 3 | 0.0200% |
| 4 | 0.0180% |
| 5 | 0.0160% |
| 6 | 0.0140% |
| 7 | 0.0130% |
| 8 | 0.0130% |
| 9 | 0.0130% |
| 10 | 0.0140% |
| 11 | 0.0150% |
| 12 | 0.0170% |
| 13 | 0.0220% |
| 14 | 0.0240% |
| 15 | 0.0270% |
| 16 | 0.0280% |
| 17 | 0.0280% |
| 18 | 0.0310% |
| 19 | 0.0290% |
| 20 | 0.0310% |
| 21 | 0.0320% |
| 22 | 0.0330% |
| 23 | 0.0330% |
| 24 | 0.0350% |
| 25 | 0.0370% |
| 26 | 0.0370% |
| 27 | 0.0390% |
| 28 | 0.0410% |
| 29 | 0.0410% |
| 30 | 0.0440% |
| 31 | 0.0470% |
| 32 | 0.0490% |
| 33 | 0.0560% |
| 34 | 0.0600% |
| 35 | 0.0650% |
| 36 | 0.0700% |
| 37 | 0.0770% |
| 38 | 0.0830% |
| 39 | 0.0950% |
| 40 | 0.1000% |
| 41 | 0.1140% |
| 42 | 0.1240% |
| 43 | 0.1360% |
| 44 | 0.1480% |
| 45 | 0.1630% |
| 46 | 0.1760% |
| 47 | 0.1910% |
| 48 | 0.2060% |
| 49 | 0.2270% |
| 50 | 0.2500% |
| 51 | 0.2670% |
| 52 | 0.3070% |
| 53 | 0.3290% |
| 54 | 0.3520% |
| 55 | 0.3870% |
| 56 | 0.4220% |
| 57 | 0.4510% |
| 58 | 0.5320% |
| 59 | 0.5530% |
| 60 | 0.6210% |
| 61 | 0.6750% |
| 62 | 0.7220% |
| 63 | 0.8050% |
| 64 | 0.8850% |
| 65 | 0.9490% |
| 66 | 1.0620% |
| 67 | 1.1680% |
| 68 | 1.3350% |
| 69 | 1.4550% |
| 70 | 1.6190% |
| 71 | 1.8690% |
| 72 | 2.0560% |
| 73 | 2.2790% |
| 74 | 2.5290% |
| 75 | 2.8890% |
| 76 | 3.1650% |
| 77 | 3.5480% |
| 78 | 3.9670% |
| 79 | 4.5270% |
| 80 | 5.0610% |
| 81 | 5.6510% |
| 82 | 6.4120% |
| 83 | 7.1300% |
| 84 | 7.7680% |
| 85 | 8.7080% |
| 86 | 9.4530% |
| 87 | 10.8600% |
| 88 | 11.5230% |
| 89 | 12.6230% |
| 90 | 14.3900% |
| 91 | 15.5990% |
| 92 | 16.7870% |
| 93 | 18.0690% |
| 94 | 19.1630% |
| 95 | 20.0520% |
| 96 | 20.6720% |
| 97 | 21.2450% |
| 98 | 21.7030% |
| 99 | 20.8010% |
| 100 | 23.7430% |

**Supplementary Table S9** Base-case costs and health outcomes under ATT weighting.

|  | EDSL | EDCSI | Increment |
| --- | --- | --- | --- |
| **Costs** | **¥27,754.95** | **¥28,686.14** | **-¥931.19** |
| Drug costs | ¥1,960.00 | ¥2,493.12 | -¥533.12 |
| Administration costs | ¥0.00 | ¥226.97 | -¥226.97 |
| Renal function monitoring costs | ¥25.57 | ¥25.57 | ¥0.00 |
| Hospitalization costs | ¥18,091.89 | ¥18,205.38 | -¥113.49 |
| Post-stroke costs | ¥7,677.49 | ¥7,735.10 | -¥57.61 |
| **QALYs** | **8.74** | **8.33** | **0.41** |
| Health status utility | 8.81 | 8.42 | 0.39 |
| Disutility | -0.07 | -0.09 | 0.02 |
| **ICER** | **-¥2,260.24** | | |
| **Result** | **More Effective & Less Costly** | | |

**Supplementary Table S10** Cost-effectiveness outcomes across alternative time horizons under ATT weighting.

|  | 90 days | 5 years | 10 years | 20 years | 30 years |
| --- | --- | --- | --- | --- | --- |
| Incremental costs（EDSL-EDCSI） | -¥927.75 | -¥962.27 | -¥959.10 | -¥943.02 | -¥932.46 |
| Incremental QALYs（EDSL-EDCSI） | 0.03 | 0.16 | 0.27 | 0.38 | 0.41 |
| **ICER** | **-¥34,033.77** | **-¥5,859.04** | **-¥3,614.95** | **-¥2,499.96** | **-¥2,277.65** |
| **Results** | All are "More Effective & Less Costly". | | | | |

**
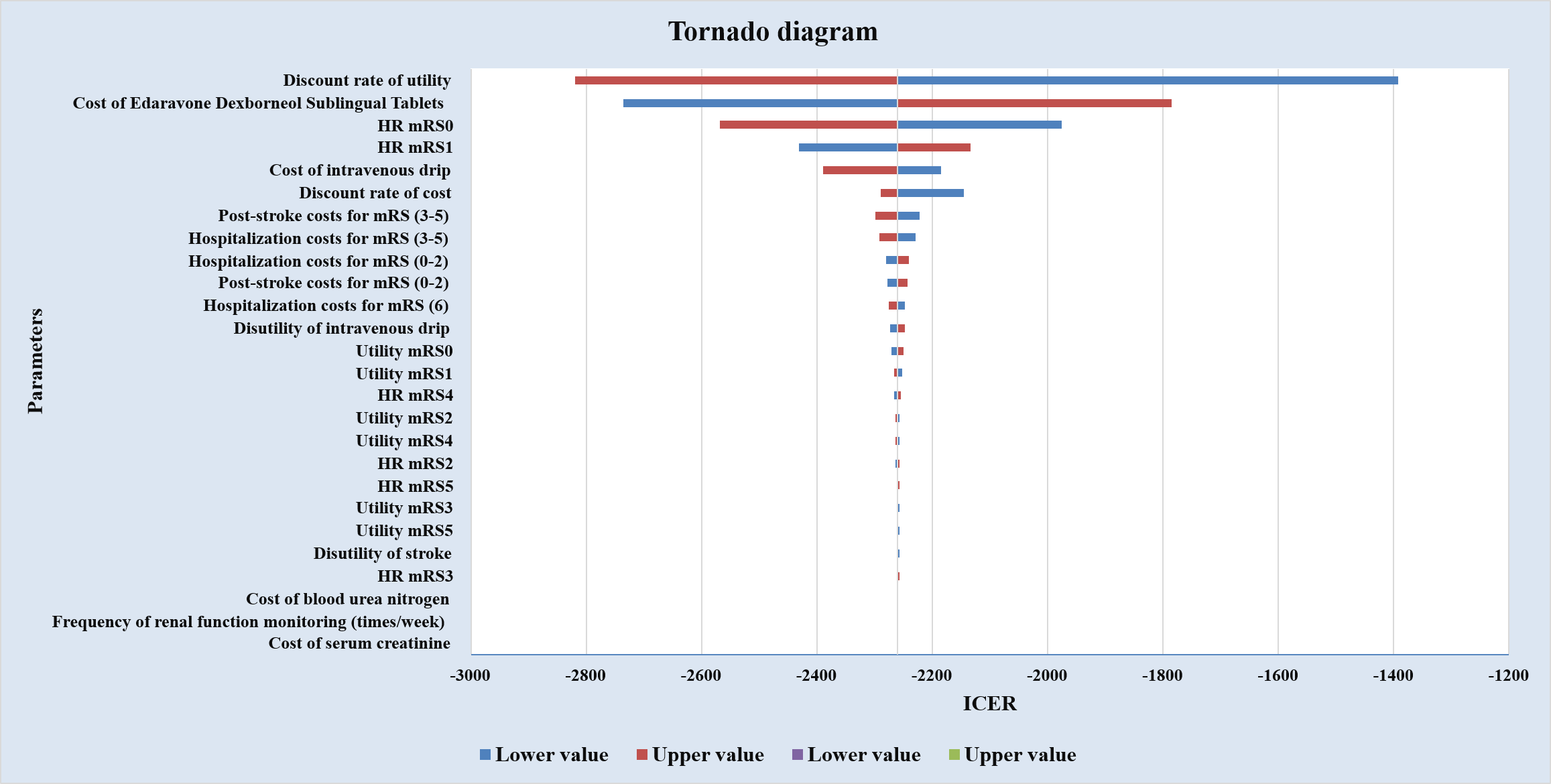
****Supplementary Figure S1** Tornado diagram under ATT weighting.

| 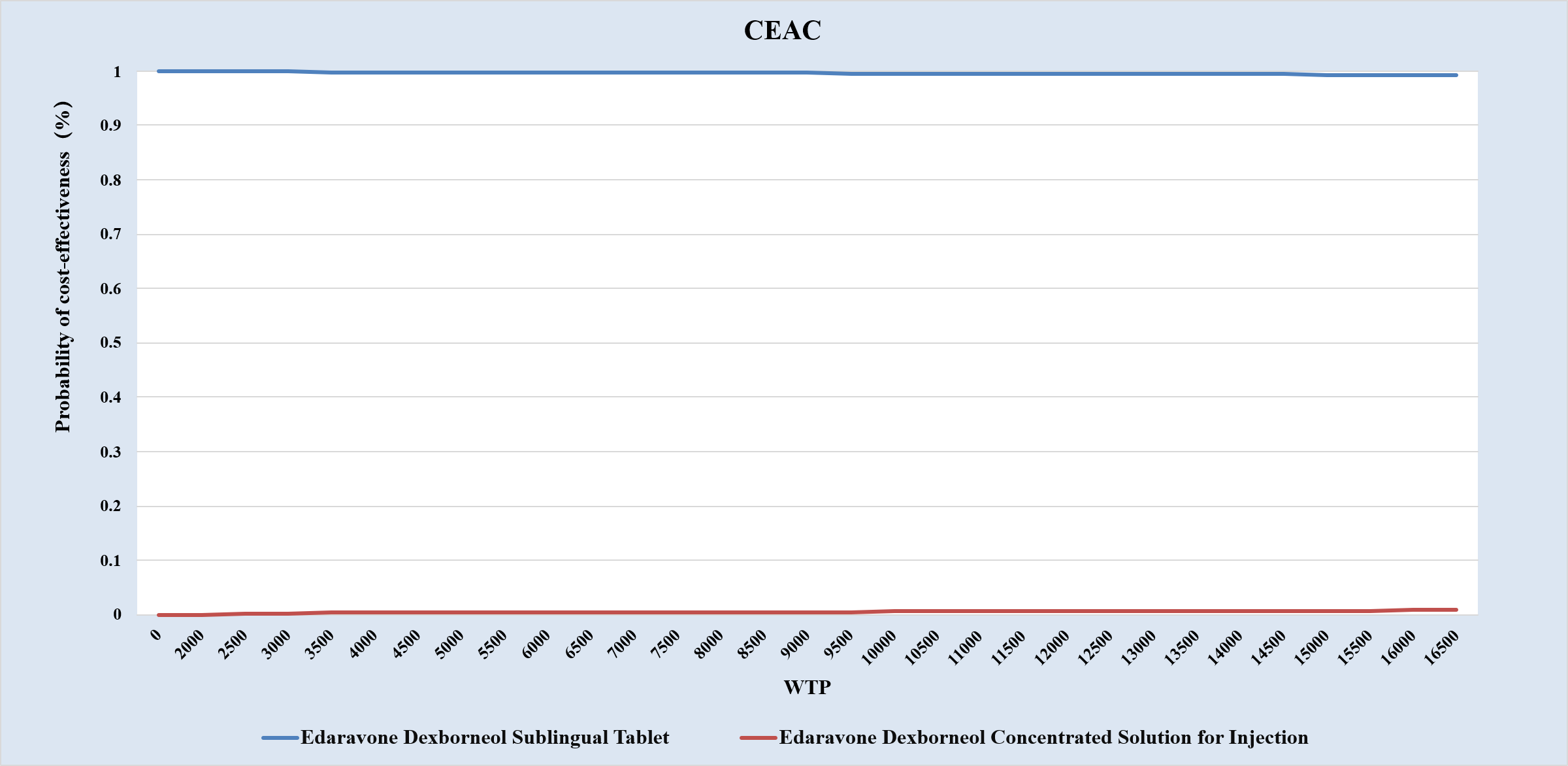A | 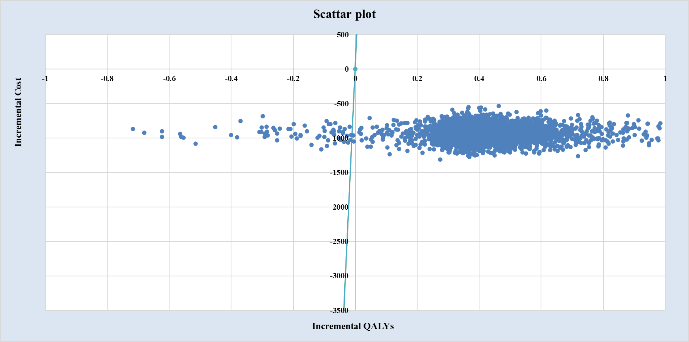B |
| --- | --- |
| **Supplementary Figure S2** Probabilistic sensitivity analysis results. A CEAC generated under ATT weighting, B ICER scatterplot under ATT weighting. | |
